# Sildenafil dosed concomitantly with bosentan for adult pulmonary arterial hypertension in a randomized controlled trial

**DOI:** 10.1186/s12872-017-0674-3

**Published:** 2017-09-06

**Authors:** Carmine Dario Vizza, Pavel Jansa, Simon Teal, Theresa Dombi, Duo Zhou

**Affiliations:** 1 Unita’ di Ipertensione Polmonare, Dipartimento di Scienze Respiratorie e Cardiovascolari, Universita’ degli Studi di Roma La Sapienza, Viale del Policlinico, 155 Rome, Italy; 2 Charles University, Ovocný trh 3-5, 116 36 Praha 1, Prague, Czech Republic; 3 Pfizer Limited, Tadworth, Berlin, UK; 4 Pfizer Inc, Eastern Point Rd, Groton, CT 06340 USA; 5 Pfizer Inc, 4 E Carol Ave, Burlingame, New York, CA 94010 USA

**Keywords:** Sildenafil - Bosentan - pulmonary hypertension - randomized controlled trial -exercise test, Combination therapy

## Abstract

**Background:**

Few controlled clinical trials exist to support oral combination therapy in pulmonary arterial hypertension (PAH).

**Methods:**

Patients with PAH (idiopathic [IPAH] or associated with connective tissue disease [APAH-CTD]) taking bosentan (62.5 or 125 mg twice daily at a stable dose for ≥3 months) were randomized (1:1) to sildenafil (20 mg, 3 times daily; *n* = 50) or placebo (*n* = 53). The primary endpoint was change from baseline in 6-min walk distance (6MWD) at week 12, assessed using analysis of covariance. Patients could continue in a 52-week extension study. An analysis of covariance main-effects model was used, which included categorical terms for treatment, baseline 6MWD (<325 m; ≥325 m), and baseline aetiology; sensitivity analyses were subsequently performed.

**Results:**

In sildenafil versus placebo arms, week-12 6MWD increases were similar (least squares mean difference [sildenafil–placebo], −2.4 m [90% CI: –21.8 to 17.1 m]; *P* = 0.6); mean ± SD changes from baseline were 26.4 ± 45.7 versus 11.8 ± 57.4 m, respectively, in IPAH (65% of population) and −18.3 ± 82.0 versus 17.5 ± 59.1 m in APAH-CTD (35% of population). One-year survival was 96%; patients maintained modest 6MWD improvements. Changes in WHO functional class and Borg dyspnoea score and incidence of clinical worsening did not differ. Headache, diarrhoea, and flushing were more common with sildenafil.

**Conclusions:**

Sildenafil, in addition to stable (≥3 months) bosentan therapy, had no benefit over placebo for 12-week change from baseline in 6MWD. The influence of PAH aetiology warrants future study.

**Trial registration:**

ClinicalTrials.gov NCT00323297 (registration date: May 5, 2006).

**Electronic supplementary material:**

The online version of this article (10.1186/s12872-017-0674-3) contains supplementary material, which is available to authorized users.

## Background

Pulmonary arterial hypertension (PAH) is a progressive, ultimately fatal disease [1, 2]. Approved PAH-specific therapies target 3 main biochemical pathways, offering opportunities for treatment with prostacyclin analogues; soluble guanylate cyclase (sGC) stimulators (riociguat [3]) or phosphodiesterase type 5 (PDE5) inhibitors (sildenafil and tadalafil); and endothelin receptor antagonists (ETRAs; bosentan, ambrisentan, and macitentan), respectively [1, 2]. Individually, these therapies improve clinical outcomes in patients with PAH in randomized controlled studies [4]. However, few controlled studies exist to support combination therapy.

International guidelines recommend sequential concomitant therapy, including (in any order) an ETRA, a PDE5 inhibitor, and a prostanoid when clinical response to initial monotherapy is inadequate [1, 4, 5] because of the possibility of additive/synergistic effects [6], despite limited supporting data. In meta-analysis of randomized controlled trials, dual concomitant therapy showed modest improvement in exercise capacity versus monotherapy (~22–25 m) in short-term (12–16 week) studies [7, 8]. Concomitant therapy with oral agents (eg, sildenafil and bosentan) may appeal to patients more than concomitant therapy with intravenous, subcutaneous, or inhaled prostanoids; however, few controlled trials have assessed oral concomitant therapy in patients with PAH. The results of a phase 2, open-label, noncomparative study (COMPASS-1) found acute reduction of pulmonary vascular resistance following the addition of a single dose of sildenafil to stable bosentan therapy [9]; this therapy was well tolerated in a 12-week, phase 4, open-label study (COMPASS-3) of a bosentan-based stepped approach with sildenafil in severe PAH [10, 11].

This was a 12-week evaluation of the safety and efficacy of oral sildenafil or placebo when used in addition to background oral bosentan therapy at a stable dose in patients with PAH. We assessed whether concomitant therapy would produce a greater improvement in exercise capacity, as assessed by 6-min walk distance (6MWD). Patients also could continue in a 52-week extension study.

## Methods

### Study design

A 12-week, multicentre, multinational, randomized, double-blind study (Part A) was conducted between September 2006 and August 2012 at 29 sites in 10 countries in patients with PAH who were receiving treatment with bosentan at a stable dose for ≥3 months to evaluate the additive effect of sildenafil therapy. The 52-week open-label extension study (Part B) continued until August 2013. The study was managed by Pfizer Inc.

Patients were randomly assigned (via interactive voice-response system incorporating a central randomization and drug supply scheme) in a 1:1 ratio to sildenafil (20 mg three times daily [TID]) or placebo for 12 weeks, administered in addition to their existing bosentan therapy (per standard of care). Patients and investigators were blinded to treatment. Randomization was intended to be stratified by baseline 6MWD (<325 m or ≥325 m) and aetiology (idiopathic/heritable PAH [IPAH/HPAH] or other). However, after blinding was broken, it was realized that only baseline 6MWD stratification had occurred. For the open-label extension, patients who completed 12 weeks of double-blind phase received sildenafil 20 mg TID in addition to bosentan for an additional 52 weeks. The use of the dosage of 20 mg TID was dictated by its approval after the SUPER-1 study [12, 13].

The coordinating ethics committee that approved the study was Comitato Etico Azienda Policlinico Umberto I (Universita’ degli Studi di Roma La Sapienza, Viale del Policlinico, 155, Roma 00155; reference number 1080/2006). At each of the 29 study centers, local institutional review boards or independent ethics committees additionally approved the trial protocol according to local and country specific guidelines. Written informed consent was obtained from each patient.

### Patients

Inclusion criteria for enrolment were adults (**≥**18 years) with PAH (mean pulmonary artery pressure ≥ 25 mmHg; pulmonary capillary wedge pressure of <15 mmHg at rest) confirmed by right heart catheterization within the previous 3 years and receiving treatment with bosentan (62.5 or 125 mg twice daily) at a stable dose for ≥3 months. PAH was idiopathic, heritable, or associated with connective tissue disease (CTD; restricted in some countries to scleroderma), or surgical repair (≥5 years previously) of septal defect (United States only). World Health Organization (WHO) functional class (FC) before initiation of bosentan therapy was III/IV (Australia and United States), III (Czech Republic, France, Germany, Greece, Italy, United Kingdom), or any FC (Israel and Taiwan). Baseline 6MWD was ≥100 and ≤450 m. Patients were required to prevent pregnancy.

Exclusion criteria were acutely decompensated heart failure within 30 days before randomization; left ventricular (LV) ejection fraction of <45% or LV shortening fraction of <0.2 within 3 months before randomization; congenital heart disease (unless meeting US inclusion criteria); history of myocardial infarction, stroke, or atrial septostomy within 6 months before randomization; uncontrolled brady- or tachy-arrhythmias, placement of pacemakers/implantable defibrillators <60 days before randomization; history of verified pulmonary embolism; history of chronic/restrictive lung disease (eg, COPD or scleroderma) with TLC <60% and/or FEV1 ≤ 80% predicted within 30 days of randomization; change of dose/class of standard background PAH therapy (ie, oxygen, calcium channel blockers, digoxin, diuretics) within 30 days (except oral anticoagulant therapy to maintain international normalized ratio within the therapeutic range); current chronic PAH-specific therapy (eg, prostacyclin, PDE5 inhibitors, ETRAs other than bosentan), nitrates/nitric oxide donors including nicorandil, or any potent cytochrome P450 3A4 (CYP3A4) inhibitors [eg, cyclosporin A, glibenclamide]); congenital heart disease (unless fulfilling US inclusion criteria), pulmonary hypertension due to thromboembolism, HIV, or schistosomiasis; previous failure on sildenafil or bosentan (defined as no evidence of clinical improvement and, on discontinuation, no worsening in symptoms/clinical status**)**; impaired renal function (serum creatinine >2.5× upper limit of normal [ULN]); and severe hepatic impairment (alanine or aspartate transaminase >3× ULN) or portopulmonary hypertension.

### Outcome measures and statistical considerations

At all post-baseline visits in the double-blind study (weeks 4, 8, and 12 or end of treatment) and at weeks 28, 40, 52, and 64 of the extension (but not at week-16 visit), 6MWD, WHO FC, [14] and Borg dyspnoea score (0 [no breathlessness] to 10 [maximum breathlessness]) [15, 16] were assessed. Clinical worsening (death, lung transplantation, hospitalization due to pulmonary hypertension, or clinical deterioration of PAH requiring additional therapy) was assessed at weeks 4, 8, and 12. In case of clinical worsening, the need to add a new specific treatment during the open-label phase of the study was left to each investigator following the local strategy.

Survival was assessed at week 64, including patients who had discontinued treatment. Blood samples were collected for analysis of plasma sildenafil and bosentan concentrations and metabolites on day 1 and week 12, with additional samples collected near trough time points at weeks 4 and 8, and for analysis of brain natriuretic peptide (BNP) and N-terminal pro-BNP at weeks 4, 12, and 64. The 6MWD was to be performed as close to trough levels of sildenafil and peak levels of bosentan as possible.

The primary endpoint was the treatment difference in change from baseline in 6MWD at week 12 (intent-to-treat population). The analysis of covariance (ANCOVA) main-effects model was used, including categorical terms for treatment, baseline 6MWD (<325 m; ≥325 m), and baseline aetiology; missing values were imputed using last-observation-carried-forward method; the same approach was used to assess week-64 data. To support interpretation of the primary analysis, sensitivity analyses were performed, including an ANCOVA on the per-protocol population, nonparametric analysis (stratified Wilcoxon test [Van Elteren]), and ANCOVA using multiple imputation approaches (informative missing, missing at random) for missing week-12 data.

There were several secondary and tertiary endpoints. Potential heterogeneity of the treatment effect in the primary endpoint across different levels of each stratification factor was investigated in separate ANCOVA models that included categorical terms of treatment effect, baseline 6MWD, and aetiology with adjustment of interaction terms “treatment*baseline walk distance” or “treatment*aetiology.” Because of a randomization stratification error, the population was stratified by baseline 6MWD only (not by aetiology). Therefore, for change from baseline 6MWD at week 12, statistical analyses for treatment comparison with adjustment of baseline 6MWD (the actual stratification factor) without adjustment of baseline aetiology were performed to evaluate the impact of the randomization stratification error. Change from baseline in 6MWD at week 12 was assessed in an exploratory post-hoc assessment in groups stratified by pre-randomization bosentan treatment duration (≤1 vs >1 y); ANCOVA analyses were performed including treatment, baseline 6MWD, and aetiology as well as the interaction term “treatment*prior bosentan duration.”

For WHO FC, Borg dyspnoea score, and time to clinical worsening, statistical analysis was to be conducted using a step-down procedure contingent upon statistical significance of the primary endpoint; otherwise, simple summary statistics are presented. Summary statistics are provided for the tertiary endpoints of change from baseline in BNP and pro-BNP. Pharmacokinetic analyses were completed for sildenafil and bosentan to investigate potential drug-drug interactions in the study population. Adverse events (AEs), monitored throughout the study, were coded using Medical Dictionary for Regulatory Activities (v15.0) and assessed for severity and relation to treatment.

The estimated sample size was based on the primary endpoint, change from baseline in 6MWD at week 12. Assuming a mean 30-m treatment difference for the sildenafil plus bosentan arm versus placebo plus bosentan arm and a standard deviation of 60 m (from SUPER-1 study assessing sildenafil monotherapy in adults with PAH [12]), a sample size of 51 patients per arm was required to detect a treatment difference with 80% power at 1-sided significance level of 0.05. Assuming a 15% dropout rate between screening and randomization and a 4% dropout rate after randomization (SUPER-1 [12]), approximately 128 patients were to be screened to ensure approximately 106 randomized and 102 evaluable patients in 2 treatment arms.

### Results

**Fig. 1:**
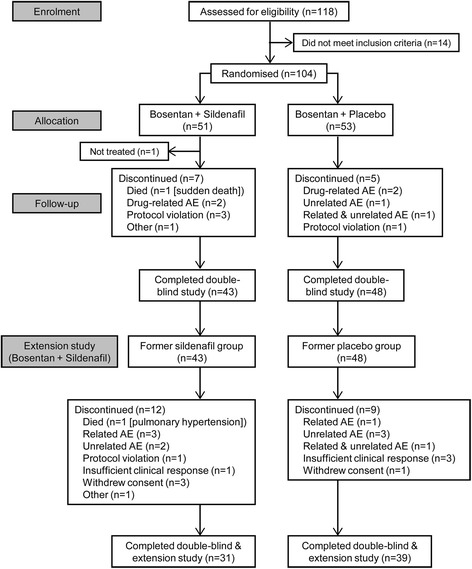
Patient disposition (CONSORT diagram). AE=adverse event

Of 104 randomized patients, 103 were treated in the double-blind study and 91 continued in the extension (Fig. 1). Most patients were women, and 34% and 65% were WHO FC II and III, respectively; the randomization stratification error caused an imbalance in baseline 6MWD between treatment groups among the 4 strata (aetiology and 6MWD; Table 1). The most common concomitant therapies in the sildenafil and placebo groups were furosemide (58% and 47%, respectively), warfarin/warfarin sodium (36% and 28%), spironolactone (32% and 32%), paracetamol (20% and 32%), and allopurinol (22% and 23%). Among patients with CTD 7/15 (46%) of the patients in the sildenafil group and 12/21(57%) in the placebo group were treated with prednisone or prednisolone. More patients in the placebo group had current cardiac disorders (19 [36%]; sildenafil, 14 [28%]), whereas more patients in the sildenafil group had current respiratory/thoracic/mediastinal disorders (25 [50%]; placebo, 15 [28%]).

**Table 1:** Baseline demographics and clinical characteristics

	Placebo (*n* = 53)	Sildenafil (*n* = 50)
Men, n (%)	12 (23)	13 (26)
Age, mean ± SD, y	56.9 ± 14.1	55.2 ± 15.1
Ethnicity, n (%)		
White	45 (85)	44 (88)
Asian	2 (4)	4 (8)
Other or unspecified	6 (11)	2 (4)
Primary diagnosis, n (%)		
IPAH/HPAH	32 (60)	35 (70)
Duration since diagnosis, median (range), y	1.3 (0.3–10.5)	1.5 (0.4–19.3)
APAH-CTD	21 (40)	15 (30)
Duration since diagnosis, median (range), y	2.0 (0.3–8.1)	1.5 (0.3–7.2)
Scleroderma	19	10
Other^a^	2	5
Bosentan treatment		
Dosage, n (%)		
62.5 mg BID	3 (6)	2 (4)
125 mg BID	49 (93)	47 (94)
Other or missing	1 (2)	1 (2)
Duration, median (range), mo	11.4 (3.1–90.9)	11.2 (3.2–65.3)
≤ 1 y, n (%)	27 (51)	26 (52)
> 1 y, n (%)	26 (49)	24 (48)
6MWD, mean ± SD, m	350.4 ± 87.6	354.4 ± 73.1
<325 m, n (%)	17 (32)	15 (30)
≥325 m, n (%)	36 (68)	35 (70)
Strata (aetiology, baseline 6MWD), n (%)		
IPAH/HPAH, <325 m	7 (13)	10 (20)
IPAH/HPAH, ≥325 m	25 (47)	25 (50)
APAH-CTD, <325 m	10 (19)	5 (10)
APAH-CTD, ≥325 m	11 (21)	10 (20)
WHO functional class, n (%)		
II	15 (28)	20 (40)
III	38 (72)	29 (58)
IV	0	1 (2)
Borg dyspnoea score, mean ± SD, median (range)	4.2 ± 1.9 4.0 (0.5–8.0)	4.1 ± 2.3 4.0 (0–8.0)
mPAP, mean ± SD, mmHg	44.9 ± 13.3	46.9 ± 12.5

*6MWD* 6-min walk distance, *APAH-CTD* connective tissue disease-associated PAH, *IPAH/HPAH* idiopathic/heritable PAH, *mPAP* mean pulmonary artery pressure, *PAH* pulmonary arterial hypertension, *WHO* World Health Organization

^a^Includes (*n* = 1 each) mixed connective tissue disease and Sharp syndrome in the placebo group; and CREST syndrome, rheumatoid arthritis, Sharp syndrome, Sjögren syndrome, and Takayasu’s disease in the sildenafil group

**Fig. 2:**
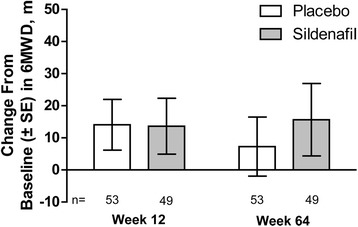
Mean (±SE) change from baseline to week 12 and week 64 in 6MWD. Last observations were carried forward. Patients are shown by their randomization in the 12-week double-blind study; however, in the 52-week open-label extension (after week 12), all patients received sildenafil and bosentan concomitant therapy. 6MWD=6-minute walk distance

The change from baseline in 6MWD at week 12 was similar between the sildenafil (13.6 m) and placebo (14.1 m) arms (LS mean difference for sildenafil – placebo, −2.4 m [90% CI: –21.8 to 17.1 m]; *P* = 0.6; Fig. 2). Per-protocol (*n* = 42 patients/arm) and other sensitivity analyses produced similar results (data not shown). An improvement of ≥30 m was experienced by 34% of sildenafil-treated (*n* = 17) and 34% of placebo-treated (*n* = 18) patients; a > 30-m worsening was seen in 10% (*n* = 5) and 11% (*n* = 6), respectively. At week 64, the change from baseline in 6MWD was numerically improved with concomitant sildenafil and bosentan therapy (Fig. 2).

**Fig. 3:**
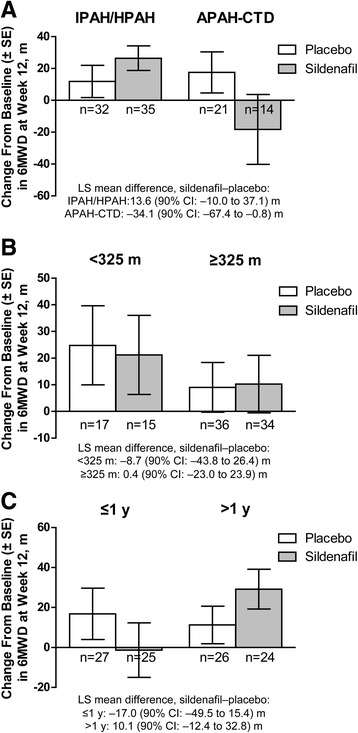
Mean (±SE) change from baseline to week 12 in 6MWD by aetiology (**a**), baseline 6MWD (**b**), and prior bosentan therapy duration (**c**). Last observations were carried forward. 6MWD=6-minute walk distance; APAH-CTD=connective tissue disease-associated PAH; IPAH/HPAH=idiopathic/heritable PAH; LS=least squares; PAH=pulmonary arterial hypertension

Because there was no statistically significant treatment effect in the primary week-12 6MWD analysis, secondary and tertiary endpoints were considered exploratory; simple summaries are provided. The change from baseline in 6MWD at week 12 differed by aetiology and by pre-randomization duration of bosentan exposure (Fig. 3). Sildenafil-treated patients with APAH-CTD had a decline in 6MWD, as did patients with ≤1 year of bosentan exposure at enrolment, whereas placebo-treated patients had improved 6MWD.

The median change in Borg dyspnoea score, BNP and pro-BNP values, and shifts from baseline in WHO FC were similar between treatment arms **(**Table 2 **)**. Population pharmacokinetic results showed concomitant administration of bosentan resulted in a 72.69% (95% CI, 66.30%–77.76%) decrease in sildenafil exposure, while concomitant administration of sildenafil produced a 19.66% (95% CI, 9.83%–30.76%) increase in bosentan exposure.

**Table 2:** Secondary and tertiary efficacy endpoints

	Change from baseline at week 12		Change from baseline at week 64		Change from week 12 to week 64	
Endpoint	Placebo (*n* = 53)	Sildenafil (*n* = 50)	Placebo (*n* = 53)	Sildenafil (*n* = 50)	Placebo (*n* = 48)	Sildenafil (*n* = 43)
Borg dyspnoea score (LOCF)						
n	53	49	53	49	47	36
Median change (range)	0 (−3, 6)	0 (−6, 2)	0 (−3, 7)	0 (−4, 4)	0 (−7, 8)	0 (−2, 4)
WHO functional class (LOCF), n (%)						
Worsened 1 class	1 (2)	0 (0)	4 (8)	5 (10)	4 (8)	8 (19)
No change	45 (85)	39 (78)	34 (64)	34 (68)	34 (71)	28 (65)
Improved 1 class	7 (13)	10 (20)	15 (28)	10 (20)	9 (19)	3 (7)
Died	0	1 (2)	0	1 (2)	0	1 (2)
Missing	0	0	0	0	1 (2)	3 (7)
BNP						
n	35	33	24	18	21	21
Median change (min, max), pg/mL	8.0 (−217.5, 254.4)	−1.0 (−436.5, 268.7)	3.0 (−125.4, 738.0)	34.3 (−618.2, 1141.8)	−6.1 (−166.9, 652.0)	20.1 (−643.7, 1218.8)
N-terminal pro-BNP						
n	14	19	9	10	9	12
Median change (min, max), pg/mL	14.3 (−2227.0, 600.0)	−94.1 (−1277.0, 221.0)	7.8 (−1761.0, 561.3)	−121.7 (−895.3, 5519.4)	55.0 (−1029.0, 466.0)	−13.2 (−459.4, 5656.2)

*BNP* brain natriuretic peptide, *LOCF* last observation carried forward, *WHO* World Health Organization

In the double-blind phase, 2 patients in each arm had clinical worsening events (hospitalization due to PAH); 1 additional patient in the sildenafil arm died (sudden death). In the extension study, the Kaplan-Meier estimate of 1-year survival from the time of randomization was 96% (90% CI, 88%–99%) for both the sildenafil and placebo arms and 96% (90% CI, 87%–99%) from the start of sildenafil treatment (delay of 12 weeks in patients randomized to placebo).

In the double-blind phase, treatment-related AEs, predominantly mild to moderate, were more common in sildenafil-treated patients. No unexpected AEs occurred **(**Table 3 **)**. Two patients (sildenafil arm) had serious treatment-related AEs (acute coronary syndrome superimposed on pre-existing 3-vessel coronary disease and underlying diabetes mellitus/hypertension, in whom a possible contributory role of sildenafil could not be excluded because of temporal association in 1 patient; the second was hypoxia deemed related by the investigator but not the sponsor). Two patients from the sildenafil group died during treatment (1 sudden death [double-blind phase] and 1 pulmonary hypertension [open-label phase]); 6 patients died during follow-up (sildenafil: pancreatic neoplasm, arrhythmia; placebo: pulmonary hypertension [*n* = 3] and sudden cardiac death).

**Table 3:** Adverse events

Adverse events (AEs)^a^	Placebo (*n* = 53)		Sildenafil (*n* = 50)	
	All causality	Treatment related	All causality	Treatment related
Patients with AEs, n (%)	41 (77)	13 (25)	34 (68)	17 (34)
Patients with serious AEs, n (%)	12 (23)	0	9 (18)	1 (2)
AEs				
Headache	5 (9)	3 (6)	6 (12)	6 (12)
Flushing	1 (2)	1 (2)	5 (10)	5 (10)
Diarrhoea	3 (6)	1 (2)	5 (10)	3 (6)
Nasopharyngitis	5 (9)	0	4 (8)	0
Vertigo	0	0	3 (6)	0
Vision blurred	0	0	3 (6)	2 (4)
Oedema, peripheral	2 (4)	1 (2)	3 (6)	1 (2)
Pulmonary arterial hypertension	5 (9)	0	2 (4)	0
Back pain	4 (8)	0	1 (2)	0
Bronchitis	4 (8)	0	1 (2)	0
Upper respiratory tract infection	3 (6)	0	1 (2)	0
Nausea	4 (8)	2 (4)	0	0

^a^Adverse events in ≥5% of patients in either treatment group during double-blind treatment with bosentan + placebo or bosentan + sildenafil

## Discussion

In this multinational, double-blind study, the change from baseline in 6MWD at week 12 (primary endpoint) was similar between the sildenafil and placebo arms in patients with PAH receiving bosentan at a stable dose for ≥3 months. Subanalyses suggested that the 6MWD response differed by PAH aetiology and pre-randomization duration of bosentan exposure. Changes in Borg dyspnoea scores, WHO functional class, and BNP and pro-BNP, as well as the incidence of clinical worsening, were similar between the 2 arms. No new or unexpected AEs occurred with sildenafil and bosentan concomitant treatment. The disappointing efficacy results were not explained by the minor randomization stratification error that resulted in slight imbalances in PAH aetiology between the 2 arms or by patient baseline comorbidities (data not shown). As discussed below, we suggest that the main reasons for the negative findings may be that patients were not truly stable on bosentan therapy at the time of randomization to sildenafil or placebo and that patients did not receive sufficient sildenafil exposure because of the drug-drug interaction with bosentan.

In our study, patients who received placebo in addition to bosentan therapy continued to improve throughout the 12-week double-blind phase, which likely affected the ability to attain a significant treatment difference in 6MWD (primary endpoint). Similarly, the nonsignificant placebo-corrected treatment effect in 6MWD at week 16 in patients receiving background bosentan in the pivotal randomized, double-blind, placebo-controlled, tadalafil study [17] occurred because of improvement from baseline in the placebo arm (mean, 19 m); the improvement in 6MWD in patients receiving background bosentan plus tadalafil (mean, 40 m) was similar to that in patients receiving tadalafil monotherapy (mean, 42 m). Although improved compliance with bosentan within the controlled study or bias because of failure to blind bosentan treatment may have affected the outcome, the authors postulated that improvement in placebo patients receiving bosentan could be attributed to continued improvement with longer bosentan exposure [17]. Our study similarly contrasts with the early indication that the maximal effect of bosentan on 6MWD is achieved within the first 3–4 months of therapy [18] and supports the idea that the definition of stable therapy should be re-evaluated for future studies [17]. Although small (*n* ≤ 32), largely uncontrolled studies have shown positive effects of sildenafil and bosentan concomitant therapy on various outcomes in PAH **(**Table 4 **)**, a comparison of the results of these studies with those of the present study is difficult because of different study designs, patient populations (PAH aetiology, functional class), and dosing regimens.

**Table 4:** Studies of concomitant sildenafil and bosentan

Study	Design	Additional PAH-specific therapy?	N	IPAH/HPAH patients %	CTD patients, %	WHO or NYHA FC II/III/IV, % of patients	Age, y, mean ± SD	Sildenafil dose	Key results
Vizza et al. (current study)	Randomized, double-blind, placebo-controlled study Sildenafil or placebo added to bosentan therapy	No	103	65%	35%	34%/65%/1%	Median (range), 59 (19–83) y	20 mg TID (bosentan 62.5 mg BID [*n* = 5] or 125 mg BID [*n* = 97]; 1 patient missing data)	No significant improvement in primary endpoint of 6MWD at week 12, nor secondary endpoints of Borg dyspnea score, clinical worsening, WHO FC, BNP/proBNP Extension study: 1-year survival of 96%; no notable improvement in 6MWD
Hoeper et al. [29]	Open label, uncontrolled study Sildenafil added to bosentan (treat to goal: 6MWD of 380 m; PVO_2_ of 10.4 ml/min/kg during CPET)	1 patient also received inhaled iloprost; 1 patient with a 2-y history of IV iloprost	9	100%	0%	0%/89%/11%	39 ± 9 y	25 mg TID, then 50 mg TID after 4–12 wk. if not reaching goal (standard bosentan^†^)	^a^3 patients improved FC at 3 mo after combination therapy; 2 others improved after 6–12 mo ^a^6MWD improved after 3 mo of combination therapy, remained stable throughout 6–12 mo follow-up ^a^PVO_2_ improved after 3 mo of combination therapy (*n* = 6 patients with CPET data)
Lunze et al. [30]	Open-label, uncontrolled study Upfront combination or sildenafil added to bosentan	No	11	36%	0% (CHD, 45%; CTEPH, 9%; radiogenic, 9%)	18%/82%/0%	Median (range), 12.9 (5.5–54.7)	Mean, 2.1± 0·9 mg/kg (bosentan, 2.3 ± 0·6 mg/kg)	After median (range) follow-up of 1.1 (0.5–2.5) y, ^a^~1-FC (NYHA) improvement ^a^Increased transcutaneous O_2_ saturation and PVO_2_ (*n* = 10) ^a^Improved 6MWD (*n* = 8) ^a^Decreased mPAP (*n* = 9), resting heart rate Right ventricular systolic pressure
van Wolferen et al. [31]	Open-label, uncontrolled study Sildenafil added to bosentan (which patients were receiving for prior 1 y) for 3 mo	NS	15	60%	20% (HIV, 7%; CHD, 13%)	13%/87%/0%	45 ± 15	50 mg BID for 4 wk., 50 mg TID until 3 mo (bosentan dose NS)	^a^Decreased NT-proBNP ^a^Increased 6MWD ^a^Improved cardiac index, right ventricular ejection fraction, right ventricular mass, RVEDV:LVEDV ratio No significant improvement: stroke volume index, left ventricular ejection fraction, left ventricular mass, RVEDV, LVEDV
Mathai et al. [32]	Open-label, uncontrolled study Sildenafil added to bosentan monotherapy in ‘failing’ patients	1/13 IPAH & 5/12 CTD patients required additional therapy during study (prostacyclins)	25	52% (included anorexigen-associated)	48% (all scleroderma-associated)	I/II = 20% III/IV = 80%	IPAH, 60 ± 8 CTD, 52 ± 13	Pre-July 2005, 25 mg TID, increased to 50 mg TID after 2–3 wk.; increased to 100 mg TID if no improvement; after, 20 mg TID (patients already on higher dose continued that dose) (bosentan^†^)	1-class NYHA FC improvement in 7 of 25 pts. (IPAH, *n* = 5; CTD, *n* = 2) ^a^6MWD improvement in IPAH pts. No 6MWD improvement in CTD pts. (Note: average daily sildenafil dose higher in CTD [168 ± 82 mg/d] vs IPAH [98 ± 65 mg/d])
Porhownik et al. [33]	Open-label, uncontrolled study Sildenafil added to bosentan in patients with inadequate improvement on monotherapy	No	10	80%	20%	50%/50%/0% (before combination therapy)	49.7 (range, 24–72)	NS	^a^Mean 6MWD improved at 6 mo vs baseline value before combination therapy initiation 1-class FC improvement in 3 pts. at 3 mo after combination therapy, maintained at 6 mo
D’Alto et al. [34]	Open-label, uncontrolled study Sildenafil added to patients with clinical deterioration on bosentan monotherapy	NS	32	0%	0% (CHD, 100% [88% Eisen-menger])	NS	37.1 ± 13.7	20 mg TID (bosentan, 94% 125 mg BID, 6% 62.5 mg BID [due to edema])	^a^Improved WHO FC, 6MWD, SpO_2_ post-exercise, Borg dyspnea index, NT-proBNP No significant differences: resting SpO_2_, heart rate (resting or post-exercise), hematocrit, hemoglobin, red blood cell count, platelets, aspartate and alanine aminotransferases
Iversen et al. [35]	Randomized, double-blind, placebo-controlled, crossover study Open-label bosentan for 9 mo, with sildenafil or placebo added at 3 mo and opposite treatment last 3 mo	No	21	0%	0% (CHD, 100% [all Eisen-menger])	43%/48%/5%	42 (range, 22–68)	25 mg TID for 2 wk., then 50 mg TID for 10 wk. (or matching placebo) (bosentan, 62.5 mg BID for 2 wk., then 125 mg BID)	Trend toward 6MWD improvement with combination therapy ^a^Resting systemic O_2_ saturation No significant changes in systolic or diastolic blood pressure; systemic O_2_ saturation during exercise; NT-proBNP; NYHA FC; pulmonary, systemic or pulmonary/systemic blood flow (assess with catheterization or MRI); pulmonary or systemic vascular resistance (Note: only patients completing the trial [*n* = 19] included in analysis)
Galie et al. [36]	Randomized, double-blind, placebo-controlled study of bosentan Subset of patients receiving open-label sildenafil	No	28	61%^‡^	18%^‡^ (CHD, 17%; HIV, 4%; other, 1%)^‡^	NS	Bosentan group, 45.2 ± 17.9^‡^ Placebo group, 44.2 ± 16.5^‡^	NS (bosentan^†^; unless <40 kg, then 62.5 mg BID)	^a^PVR improved vs baseline at 6 mo No significant difference in 6MWD at 6 mo
Benza et al. [abstract] [10]	Open-label, uncontrolled study Treat to goal (6MWD ≥380 m), bosentan for 16 wk.; continue monotherapy or add sildenafil for additional 12 wk	No (patients were treatment-naïve)	100	NS	NS	NS/NS/79%/NS	56	20 mg TID (bosentan, 125 mg BID)	31% of patients reached 6MWD goal (bosentan, 16%; combination, 15%); mean improvement of 22 and 45 m vs baseline at wk. 16 and 28, respectively 23% of patients had ≥1-class FC improvement vs baseline at wk. 16; 34% at wk. 28 (*n* = 85)
McLaughlin et al. [24]	Randomized, double-blind, placebo-controlled study Bosentan or placebo added to stable dose sildenafil (≥12 mo) until first morbidity/mortality event	NS	334	NS	NS	NS	NS	≥20 mg TID (bosentan 125 mg BID)	No significant improvement in time to first morbidity/mortality event vs placebo (primary endpoint; 17% risk reduction vs placebo; *P* = 0.25) Placebo-corrected improvement of 21.8 m in 6MWD at wk. 16; NT-proBNP level over 20 mo was 23.5% lower with bosentan vs placebo; no treatment difference for other endpoints

*6MWD* 6-min walk distance, *APAH* associated PAH, *BID* twice daily, *BNP* brain natriuretic peptide, *CHD* congenital heart defect, *CPET* cardiopulmonary exercise testing, *CTD* connective tissue disease, *CTEPH* chronic thromboembolic pulmonary hypertension, *ETRA* endothelin receptor antagonist, *FC* functional class, *IPAH/HPAH* idiopathic PAH/heritable PAH, *IV* intravenous, *LVEDV* left ventricular end diastolic volume, *mPAP* mean pulmonary arterial pressure, *MRI* magnetic resonance imaging, *NS* not stated, *NT-proBNP* N-terminal pro-brain natriuretic peptide, *NYHA* New York Heart Association, *PAH* pulmonary arterial hypertension, *PDE5i* phosphodiesterase type 5 inhibitor, *PVO*_*2*_ peak oxygen consumption, *PVR* pulmonary vascular resistance, *RVEDV* right ventricular end diastolic volume, *SpO*_*2*_ transcutaneous oxygen saturation, *TID* three times daily, *WHO* World Health Organization

^a^Denotes statistically significant improvement vs baseline (also vs bosentan monotherapy [Mathai])

^†^62.5 mg BID for 4 weeks, 125 mg BID thereafter

^‡^Value is for the overall study (*n* = 185); not reported for the *n* = 28 patient subset receiving combination therapy

The pharmacokinetic analysis in the current study indicated a substantial drug-drug interaction, with sildenafil exposure decreased by 73% and bosentan exposure increased by 20% with concomitant administration. Bosentan is a CYP3A4 inducer, whereas the PDE5 inhibitor sildenafil is a CYP3A4 substrate. [19] Although sildenafil is not an inhibitor of CYP3A, it interferes with the hepatic uptake transporters OATP1B1/1B3 for which bosentan is a substrate [19]. In healthy volunteers, concomitant administration increased the bosentan plasma concentration (area under the curve) by ~50% and decreased the sildenafil plasma concentration by ~60% [20]. Similar results were described in patients with PAH treated concomitantly with these two therapies [21].

Concomitant therapy with an ETRA and a PDE5 inhibitor has shown some potential for clinical efficacy in PAH. In a randomized, double-blind study of patients (*n* = 124) who received tadalafil or placebo added to stable ambrisentan therapy for 16 weeks, patients receiving concomitant therapy had significantly improved 6MWD and significantly fewer clinical worsening events than patients receiving placebo, with no increase in AEs [22]. Furthermore, the risk of clinical failure and 24-week 6MWD were significantly improved with up-front concomitant therapy with ambrisentan and tadalafil versus monotherapy in 500 treatment-naive patients with PAH [23]. A phase 4 study (COMPASS-2), in which patients receiving sildenafil were randomized to bosentan or placebo, demonstrated a significant improvement in week-16 6MWD (exploratory analysis) with sildenafil-bosentan concomitant therapy versus sildenafil-placebo, but no significant treatment difference was observed in time to first morbidity/mortality event (primary endpoint) [24].

The 6MWD has been a primary endpoint in registration studies of all but two of the approved PAH therapies; however, some studies have noted that changes in 6MWD do not correlate with long-term outcomes [25, 26], and the clinical relevance of short-term changes is unclear [27]. Hemodynamic parameters, which were not assessed during the current study, may have provided additional insights on clinical outcomes.

Taking into account the results of studies evaluating the combination of a PDE-5i and an ERA, the variability could be explained by the pharmacokinetic interaction of some combination (bosentan-sildenafil) or the behaviour of placebo group (our study, PHIRST). Even in the absence of head to head comparison, one could speculate that the drugs in the same class are not similar and may not be interchangeable without clinical impact. Finally, it seems that the choice of the combination and the timing of intervention could have a role in the treatment strategy of PAH.

Important clinical questions on combination therapy timing remain unanswered, including whether concomitant therapy should be initial or sequential. Additional controlled trials of add-on therapy in patients with PAH are needed. The influence of PAH aetiology on efficacy outcomes with concomitant therapy and the duration of background therapy should be taken in account for the design of further study.

Although our study was placebo controlled, several factors limit the conclusion that this combination, recommended by international guidelines [5, 28], is not clinically effective. First, sample size calculations were based on observations in treatment-naïve patients receiving sildenafil monotherapy [12], and in retrospect, this level of 6MWD improvement appears optimistic. Second, patients who are already receiving effective bosentan monotherapy likely have decreased potential for improvement (ie, a ceiling effect). Finally, the enrolment was slow, and changes in therapeutic strategies could have influenced the results of the present study.

## Conclusions

In this multinational trial of predominantly WHO FC II and III PAH patients, sildenafil was well tolerated, but provided no additional benefit versus placebo on week-12 6MWD when used in addition to stable (≥3 months) bosentan therapy. Study limitations, including the definition of stable therapy, and study results demonstrating the importance of drug-drug interactions should be helpful in the design of future clinical trials of concomitant therapies for PAH.

## Additional file

Additional file 1: List of Independent Ethics Committees. A list of the independent ethics committees, sorted by country and site, for sites that screened subjects. (PDF 105 kb)

## Abbreviations

6MWD: 6-min walk distance
AE: Adverse event
ANCOVA: Analysis of covariance
APAH: Associated PAH
BID: Twice daily; BNP = brain natriuretic peptide
CHD: Congenital heart defect
CI: Confidence interval
CPET: Cardiopulmonary exercise testing
CTD: Connective tissue disease
CTEPH: Chronic thromboembolic pulmonary hypertension
ETRA: Endothelin receptor antagonist
FC: Functional class
IPAH/HPAH: Idiopathic PAH/heritable PAH
IV: Intravenous
LVEDV: Left ventricular end diastolic volume
mPAP: Mean pulmonary arterial pressure
MRI: Magnetic resonance imaging
NT-proBNP: N-terminal pro-brain natriuretic peptide
NYHA: New York Heart Association
PAH: Pulmonary arterial hypertension
PDE5i: Phosphodiesterase type 5 inhibitor
PVO_2_: Peak oxygen consumption
PVR: Pulmonary vascular resistance
RVEDV: Right ventricular end diastolic volume
SpO_2_: Transcutaneous oxygen saturation
TID: Three times daily
WHO: World Health Organization
