# Supplementary material for: Sildenafil dosed concomitantly with bosentan for adult pulmonary arterial hypertension in a randomized controlled trial
Source: BMC Cardiovasc Disord. 2017 Sep 6;17:239. doi: 10.1186/s12872-017-0674-3 (PMC5586020; doi:10.1186/s12872-017-0674-3)
Supplement: Additional file 1: — List of Independent Ethics Committees. A list of the independent ethics committees, sorted by country and site, for sites that screened subjects. (PDF 105 kb) [file 12872_2017_674_MOESM1_ESM.pdf]

The List of Independent Ethics Committees, sorted by country and site, for sites that screened subjects

Independent Ethics Committees Site Number	Name and Address of Committee
**Australia**	
1016	St. Vincent's Hospital Human Research Ethics Committee<br>Research Office Level 6 De Lacy Bldg<br>Victoria Street<br>Darlinghurst, NSW 2010
1017	Prince Charles Hospital Human Research & Ethics Committee<br>The Prince Charles Hospital<br>Rode Road<br>Chermside, QLD 4032
**Czech Republic**	
1032<br>1033	Eticka komise IKEM a FTNsP<br>Videnska 800<br>Praha 4 Krc 140 59
**France**	
1013<br>1034<br>1038	Comité de protection des<br>personnes<br><Nord Ouest IV><br>CHU Faculté de Médecine<br>Pôle Recherche<br>Lille 59045
**Germany**	
1005<br>1006<br>1007<br>1008<br>1036<br>1037<br>1042<br>1044<br>1051<br>1052	Ethik-Kommission des Fachbereichs Medizin<br>Der Justus-Liebig- Universitaet Giessen<br>Gaffkystrasse 11 c<br>Giessen 35385

**Greece**	
1040	National Ethics Committee<br>284 Mesogion Avenue<br>Athens 15562
**Israel**	
1024	Rabin MC Ethics Committee<br>Rabin MC<br>Belinson Campus<br>Petah Tikva 49100
1026	Rambam Medical Center<br>Helsinki Committee<br>POB 9602, Bat Galim<br>Haifa 31096
**Italy**	
1027	Comitato Etico Azienda Policlinico Umberto I<br>Viale del Policlinico, 155<br>Roma 00155
1028	Comitato Etico, Azienda Ospedaliera Monaldi<br>Via Leonardo Bianchi<br>Napoli 80131
**Taiwan**	
1039	National Taiwan University Hospital,<br>Research Ethics Committee<br>7 Chung Shan South Road<br>Taipei 100
**United Kingdom**	
1035	Huntingdon Local Research Ethics Committee<br>REC Office<br>Victoria House<br>Capital Park<br>Fulbourne<br>Cambridge, Cambridgeshire CB1 5XB

**United States**	
1009	BCM Office of Research<br>Institutional Review Board for Human Subjects<br>Room 600D<br>One Baylor Plaza<br>Houston, TX 77030
1018	Veterans Affair Greater Los Angeles<br>Healthcare System IRB<br>Research and Development Office<br>11301 Wilshire Boulevard<br>Building 114, Mail Code-151<br>Los Angeles, CA 90073
1021	Allegheny General Hospital<br>Institutional Review Board<br>15th Floor, South Tower<br>320 East North Avenue<br>Pittsburgh, PA 15212-4772
1029	Henry Ford Hospital<br>Institutional Review Board (IRB)<br>2799 West Grand Boulevard<br>CFP - B<br>Detroit, MI 48202
1031	Western Institutional Review Board<br>3535 Seventh Avenue, South West<br>Olympia, WA 98502
